# Ultrasound Image Enhancement Using Structure-Based Filtering

**DOI:** 10.1155/2014/758439

**Published:** 2014-06-19

**Authors:** Shyh-Kuang Ueng, Cho-Li Yen, Guan-Zhi Chen

**Affiliations:** ^1^Department of Computer Science, National Taiwan Ocean University, No. 2, Peining Road, Keelung City 202, Taiwan; ^2^Chang-Gung Memorial Hospital, No. 222, Mai-Chin Road, Keelung City 204, Taiwan

## Abstract

Ultrasound images are prone to speckle noises. Speckles blur features which are essential for diagnosis and assessment. Thus despeckling is a necessity in ultrasound image processing. Linear filters can suppress speckles, but they smooth out features. Median filter based despeckling algorithms produce better results. However, they may produce artifact patterns in the resulted images and oversmooth nonuniform regions. This paper presents an innovative despeckle procedure for ultrasound images. In the proposed method, the diffusion tensor of intensity is computed at each pixel at first. Then the eigensystem of the diffusion tensor is calculated and employed to detect and classify the underlying structure. Based on the classification result, a feasible filter is selected to suppress speckles and enhance features. Test results show that the proposed despeckle method reduces speckles in uniform areas and enhances tissue boundaries and spots.

## 1. Introduction

In ultrasound scanning, the reflected sound waves are mainly generated by tissue boundaries. Nonetheless, smaller structures in relatively homogeneous regions can also produce echoes with random phases. These echoes trigger constructive and destructive interferences and generate speckle patterns in the ultrasound images [1]. Speckles deteriorate tissue boundaries and make homogeneous regions look rough. Thus essential information for diagnosis is lost. Gaussian filters [2] can suppress speckles and enhance contrast, but they blur edges [3]. In [4], Deng and Cahill proposed an adaptive Gaussian filter to solve this problem. They adjusted the variance of the Gaussian filter during the smoothing process so that noises were reduced and edges were better preserved. Adaptive median filters are superior to Gaussian filters in despeckling [5]. However they may produce unnatural patterns in the resultant images [6, 7].

**Figure 1:**
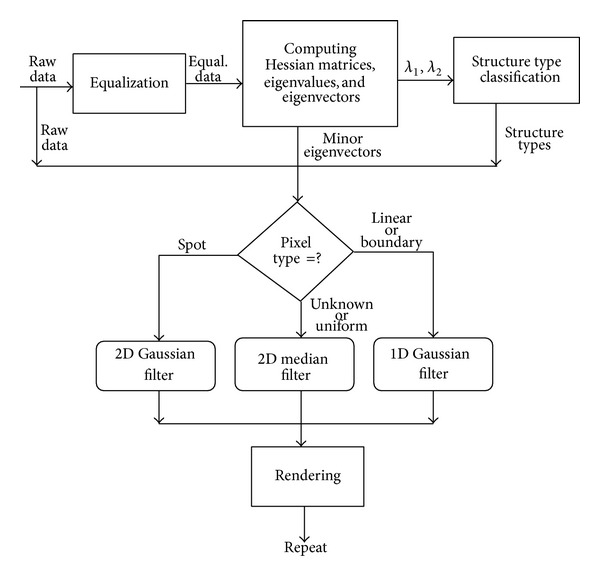
The flowchart of the proposed despeckle method.

In this paper, we propose a structure-based despeckling method for ultrasound data. In the proposed method, despeckling is divided into several stages and carried out in a pipeline manner. The flowchart of the proposed procedure is displayed in Figure 1. The despeckling method starts with equalizing the input ultrasound image. Then, at each pixel, the eigensystem of a Hessian matrix is computed to measure the strength and orientation of the local diffusion tensor. In the following step, the local diffusion tensor is used to classify the pixel into one of the following types:* linear*,* boundary*,* spot*,* uniform*, and* unknown*. In the final stage, feasible filters are adaptively selected to suppress speckles. If a pixel is spot-typed, it is filtered by using a 2D Gaussian filter. If the pixel's type is uniform or unknown, it is smoothed by using a 2D median filter. If the pixel is classified as linear- or boundary-typed, it is filtered by using a 1D Gaussian filter carried out in the direction of the minor eigenvector of the Hessian matrix. Once the despeckling process is completed, the resulted image is displayed. If the image quality does not satisfy our goal, the whole despeckling pipeline is repeated until speckles are significantly removed.

### 1.1. Related Work

In ultrasound image processing, feature preservation is usually in conflict with despeckling. To alleviate this problem, researchers utilize local information of roughness to selectively reduce speckles. In [8], a directional median (DM) filter method is proposed to reduce noise and enhance edges for ultrasound images. In their method, a 1D median filtering process is performed in various directions at each individual pixel. The filtering result is set to the maximum response of these 1D median filtering processes. Loupas et al. developed an adaptive median filter, the* AWMF* method, to suppress speckles [9]. In their method, local SNR values are used to compute the weights of the pixels inside the median filter's mask. Then these pixels are duplicated according to the computed weights. Finally the new pixel value is set to the median value of the duplicated pixels.

The diffusion of an intensity field reveals the spreading of the intensity field. The diffusion magnitude reflects the strength of the intensity variation, while the diffusion direction shows the direction of the intensity spreading. Based on these two principles, diffusion of intensity has been utilized in various filtering algorithms to enhance features and reduce noises. Abd-Elmoniem et al. propose a diffusion-based method in [10]. They compute structure matrices to estimate local coherence for ultrasound images. Then an anisotropic diffusion governing equation is iteratively solved so that fully developed speckles are suppressed while tissue structures of the resolved granularity are preserved. In another work [11], Ueng et al. utilize the eigensystems of Hessian matrices to estimate the local diffusion strengths and directions for all the voxels of medical imaging data so that tissue types can be classified. Then the tissue types are served as criteria to adjust the variance of a Gaussian filter which is employed to reduce noise and enhance region boundaries. Krissian et al. present a novel method for constructing aorta from abdominal ultrasound data [12]. They combine the Hessian and structure matrices of the intensity field to form a* descriptor* matrix at each pixel. The eigensystem of the descriptor matrix is used to detect the orientation of aorta. Then, a specialized response function is evaluated to find the cross-section boundaries of aorta. By connecting the cross-section boundaries, the aorta is constructed.

In the proposed method, local roughness and diffusion tensor are simultaneously employed for feature detection. Using this information, an elaborated classification procedure is dedicated to explore features and classify local regions into various structure types. Since ultrasound data are prone to noises, conventional medical image classification methods may produce short boundaries and scattering spots. An iterative classification refinement strategy is adopted to connect broken edges and remove tiny spots so that larger regions and longer boundaries are formed. Different filters have their pros and cons. In this work, a heterogeneous and adaptive filtering technique is used to smooth noises and preserve features. Linear and nonlinear filters are selectively utilized for suppressing speckles based on the detected structure types. Therefore, uniform regions are smoothed while boundaries are enhanced and spots are preserved.

## 2. Materials and Methods

### 2.1. Diffusion Tensor and Structure Type Classification

The diffusion tensor at a pixel can be represented by a Hessian matrix. The Hessian matrix, *H*(*x*, *y*), pixel *I*(*x*, *y*), is defined by

(1) $$\begin{array}{c} H=\left[\begin{array}{cc} I_{xx} & I_{xy}\\ I_{xy} & I_{yy} \end{array}\right]=\left[\begin{array}{cc} \frac{\partial^{2}I}{\partial x^{2}} & \frac{\partial^{2}I}{\partial x\partial y}\\ \frac{\partial^{2}I}{\partial x\partial y} & \frac{\partial^{2}I}{\partial y^{2}} \end{array}\right]. \end{array}$$

Since *H* is symmetric, its eigenvalues are real and its eigenvectors are mutually orthogonal. Let *λ*_1_ and *λ*_2_ be the eigenvalues with ||*λ*_1_|| ≥ ||*λ*_2_|| and ${\vec{v}}_{1}$ and ${\vec{v}}_{2}$ the corresponding eigenvectors. *λ*_1_ and *λ*_2_ are the major and minor eigenvalues; and ${\vec{v}}_{1}$ and ${\vec{v}}_{2}$ are the major and minor eigenvectors.

The eigensystem reflects the anisotropy and strength of the diffusion tensor. It is used to identify the local structure type [13]. In [14], Sato et al. utilized eigensystems of Hessian matrices to classify tissue types for medical imaging. The classification principles can be formulated as follows. If the magnitudes of the eigenvalues are small, the local region is relatively uniform. No feature is available. If the magnitude of the major eigenvalue is much larger than that of the minor eigenvalue, the intensity varies significantly in the direction of the major eigenvector but alters slightly in the direction of the minor eigenvector. The diffusion is anisotropic, and the underlying structure is 1-dimensional. When the magnitudes of both eigenvalues are large and roughly the same, the intensity is concentrated at the pixel. The diffusion is isotropic. The local region is a 2-dimensional spot.

**Figure 2:**
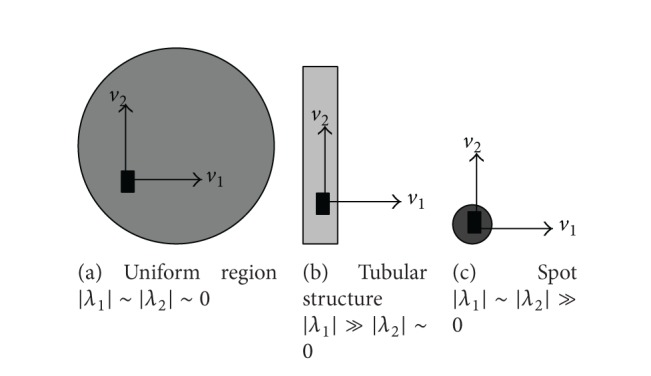
The eigenvalues of the diffusion tensor reveal the underlying structure type.

These cases are illustrated in Figure 2. In part (a), both eigenvalue magnitudes are small. The region surrounding the pixel is nearly homogeneous. In part (b), the intensity is nearly uniform along the direction of the minor eigenvector but changes abruptly in the direction of the major eigenvector. The local region is a tubular structure. In part (c), the intensity variations in both directions are high and the local region is a bright or dark spot.

### 2.2. The Structure Classification Method

As shown in Figure 2, a pixel can be classified as a part of a uniform, tubular, or spot structure-based on the eigensystem of the local diffusion tensor. The rules of this preliminary classification method are summarized in Table 1. However, there is no objective method to tell whether an eigenvalue is large or not. These rules are logically correct but not practical. In this section, a practical and efficient classification method is presented for detecting features and classifying structure types.

After the eigensystems of the local diffusion tensor have been computed at all the pixels, the maximum magnitude of the eigenvalues is searched. The maximum eigenvalue magnitude is used to normalize the eigenvalues so that their values are within −1 and 1. Then two variables *T*_1_ and *T*_2_ are computed by using the normalized eigenvalues at each pixel by

(2) $$\begin{array}{c} T_{1}=\sqrt{\lambda_{1}^{2}+\lambda_{2}^{2}},\\ T_{2}=\frac{||\lambda_{2}||+\epsilon }{||\lambda_{1}||+\epsilon }. \end{array}$$

*T*_1_ is employed to test whether there is a feature in the local region. In a homogeneous region, the magnitudes of both eigenvalues are small. Thus *T*_1_ is nearly 0. In a tubular structure, the magnitude of the major eigenvalue is large but the magnitude of the minor eigenvalue is small so that *T*_1_ is much larger than 0 and closer to 1. In a spot, both eigenvalue magnitudes are large. *T*_1_ can be greater than 1 but less than $\sqrt{2}$. *T*_2_ is used to distinguish a tubular structure from a spot. In a tubular structure, ||*λ*_1_|| is much larger than ||*λ*_2_||, and thus *T*_2_ is nearly 0. In a spot, both eigenvalue magnitudes are large and similar so that *T*_2_ is approximately 1. In computing *T*_2_, a tiny number *ϵ* is added to the denominator and the numerator to avoid* dividing by zero* and misjudgement in case both eigenvalues are nearly 0.

Then, by using *T*_1_ and *T*_2_, we define two response functions to measure the degrees of linearity and spot:

(3) $$\begin{array}{c} C_{l}=T_{1}*\left(1-T_{2}\right),\\ C_{s}=T_{1}*T_{2}. \end{array}$$

*C*_*l*_ and *C*_*s*_ are the measurements of linearity and spot, respectively. If the local area is not uniform and the ratio of the minor eigenvalue magnitude to the major eigenvalue magnitude is small, *C*_*l*_ is close to 1 and *C*_*s*_ is close to 0. On the other hand, if both eigenvalue magnitudes are significant and roughly equal, *C*_*s*_ is closer to or even larger than 1 and *C*_*l*_ is nearly 0. Now, based on *T*_1_, *C*_*l*_, and *C*_*s*_, we can detect and classify the local structure.

In the preliminary structure classification rules listed in Table 1, only three structure types are defined. In order to retrieve more structure information for the following despeckling process, we refine the classification model and categorize pixels into 5 types:* linear*,* boundary*,* spot*,* uniform*, and* unknown*. Our pixel classification algorithm works as follows.

(i) A pixel is regarded as uniform-typed, if
  (4) $$\begin{array}{c} T_{1}\leq \beta , \end{array}$$
 where *β* is a small number defined by users. In our implementation, *β* is set to 0.12.
(ii) A pixel is classified as linear-typed, if
  (5) $$\begin{array}{c} T_{1}>\beta ,\;\;C_{l}>C_{s},\;\;\lambda_{1}\leq 0. \end{array}$$
(iii) A pixel is identified as boundary-typed, if
  (6) $$\begin{array}{c} T_{1}>\beta ,\;\;C_{l}>C_{s},\;\;\lambda_{1}>0. \end{array}$$
(iv) A pixel is a spot pixel, if
  (7) $$\begin{array}{c} T_{1}\geq \alpha ,\;C_{s}>C_{l}, \end{array}$$
 where *α* is a number selected by users. Based on our experiments on filtering ultrasound and gray-level images, *α* is set to 0.45 in our implementation.
(v) Else, the pixel type is registered as unknown-typed.

If *T*_1_ is small, the intensity variation is weak and the pixel is in a uniform region. Otherwise, we check whether *C*_*l*_ dominates *C*_*s*_ or not. If so, the tendency of linearity is stronger. Then the major eigenvalue *λ*_1_ is used to identify whether the pixel is in a bright edge (local maximum) or a dark boundary (local minimum). If *λ*_1_ is negative, the region is the local maximum. The pixel is in a bright edge and regarded as linear-typed. If the major eigenvalue is positive, the pixel is the local minimum. The pixel is identified as a boundary pixel. In case that *C*_*s*_ is greater than *C*_*l*_, the tendency of spot is stronger. The pixel is regarded as spot-typed. However, to avoid misjudging a noisy region as a spot region, *T*_1_ is required to be greater than a predefined threshold *α* = 0.45. If all of these tests fail, we cannot identify the structure type, and thus the pixel is classified as unknown-typed.

#### 2.2.1. Type Classification Refinement

In ultrasound scanning, tissue boundary segments with high curvatures produce weaker echoes. Pixels in these segments have lower intensities than their neighbors and may be classified as spots or unknown structures. In order to improve the classification quality, a type refinement process is performed to reclassify pixels after the structure classification process is completed. Thus broken tubular structures can be merged and scattered spots can be assimilated into the adjacent tubular structures. The refinement algorithm works as follows.

- Keep all spot pixels and unknown-typed pixels in a queue.
- Retrieve these pixels from the queue one by one.
- For each retrieved pixel do the following.
  1. Check the two adjacent neighboring pixels, one pixel in the positive direction and the other one in the negative direction of the minor eigenvector.
  2. If any of the neighbors is a linear pixel, the pixel is reclassified as linear-typed.
  3. Else if any of the neighbors is a boundary pixel, the pixel is regarded as a boundary pixel.
  4. Else restore the pixel into the queue.
- Repeat the reclassification 3 times.

**Figure 3:**
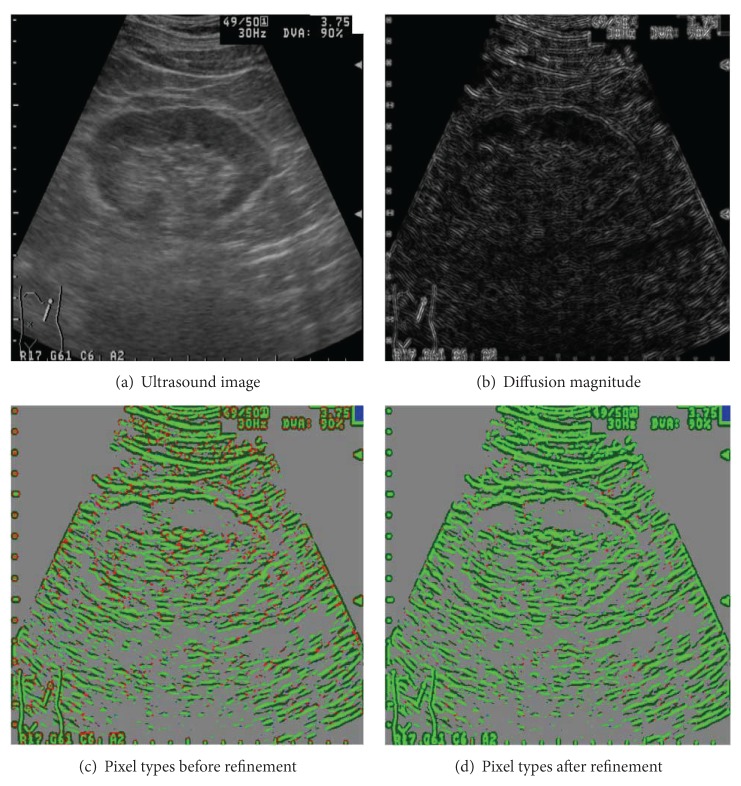
Structure type classification, (a) the ultrasound image, (b) the diffusion strength, (c) classified structure types, and (d) refined structure classification, green: linear, dark green: boundary, red: spot, blue: unknown, and grey: uniform.

A structure classification example is displayed in Figure 3. The ultrasound image is shown in part (a). In part (b), the diffusion strength, measured by using *T*_1_, is rendered. The classification results with no refinement are illustrated in part (c). The refined classification results are shown in part (d). Grey color is used to render uniform-typed pixels. Linear-typed pixels and boundary-typed pixels are rendered in green and dark-green colors. Spot-typed pixels and unknown-typed pixels are illustrated in red and blue colors. Comparing (c) and (d), we find that most sharp corners are successfully reclassified as linear or boundary structures. Spot-typed pixels and unknown-typed pixels, adjacent to linear and boundary structures, are mostly assimilated into the neighboring tubular structures.

### 2.3. The Structure-Based Despeckle Method

All filters have their pros and cons. Some filters can significantly reduce speckles, but they blur edges. Other filters preserve edges but may produce unwanted patterns. In this work, we adopt a heterogeneous despeckling strategy. Pixels of different types are smoothed by using different filters so that speckles in uniform regions are reduced and tissue boundaries and edges are preserved.

#### 2.3.1. The Filtering Strategy

We create a filter pool which includes a 1D Gaussian filter, a 2D median filter, and a 2D Gaussian filter. These filters are adaptively selected to smooth pixels according to the following principles.

- Linear-typed and boundary-typed pixels are filtered by using the 1D Gaussian filter. The standard deviation of the 1D Gaussian filter is 1. The filter mask is composed of 9 pixels. Its direction is parallel to the minor eigenvector of the target pixel.
- Uniform-typed pixels are smoothed by using the 2D median filter. The mask of the median filter is composed of 9 × 9 pixels.
- Spot-typed pixels are filtered by using the 2D Gaussian filter. The standard deviation of this 2D Gaussian filter is 1.
- Unknown-typed pixels are smoothed by using the 2D median filter.

In the classification process, bright and dark curves are classified as linear and boundary structures. For a pixel residing in a curve, its minor eigenvector ${\vec{v}}_{2}$ is tangent to the curve. To filter the pixel, a 1D stick centered at the pixel and parallel to ${\vec{v}}_{2}$ is created at first. Then the 1D Gaussian filtering process is carried out at the pixel by using the stick as the filter mask. After filtering, the intensity along the curve becomes more coherent, while the intensity contrast across the curve is increased. Thus the curve is not only preserved but also enhanced. Sharp corners, endpoints of edges, and isolated bright and dark points are classified as spot structures. Spot structures are filtered by using the 2D Gaussian filter so that small spots are blended into the surrounding areas, while large spots are preserved. Pixels residing in uniform areas are smoothed by using the 2D median filter. Therefore speckles and pepper-and-salt noises in uniform areas can be removed. At unknown-typed pixels, no obvious feature can be detected. The 2D median filter is used to filter these pixels so that thin and fuzzy structures are removed, but thick structures are preserved.

#### 2.3.2. Iterative Filtering Process

**Figure 4:**
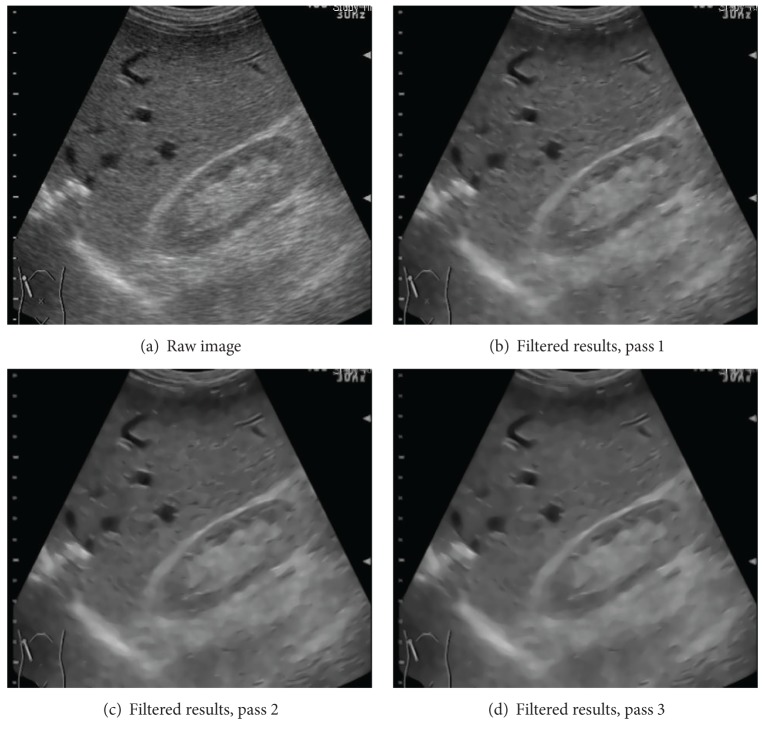
Filtered results. (a) The raw data and (b)–(d) the results of the 1st, 2nd, and 3rd filtering passes.

If the filtered image still contains significant speckles, the whole despeckling pipeline is repeated until the image quality satisfies our goal. The number of filtering passes, required to achieve the satisfied results, is data-dependent. Three filtering passes may be needed for a highly noisy image, while one filtering pass is sufficient for a relatively clean image. For ordinary ultrasound images, a 2-pass despeckling process is the most cost-effective strategy according to our experiments. An example is presented in Figure 4 to show the effectiveness of using multiple despeckling passes. The raw ultrasound image is shown in part (a), and the images produced by the 1st, 2nd, and 3rd filtering passes are displayed in parts (b)–(d). As shown in these images, speckles are removed while longer and thicker tissue boundaries are enhanced as additional despeckling passes have been carried out. The resultant image of the 3rd pass is the smoothest one, though some thin edges and small spots are eliminated.

## 3. Results and Discussion

Based on the detected structure types, the proposed despeckle method employs a 2D median filter, a 1D Gaussian filter, and a 2D Gaussian filter to suppress speckles and enhance features. It is an extension and combination of median and Gaussian filters. We compare our filtering method with a Gaussian filter, a median filter, the AWMF method [9], and the directional median (DM) filter [8]. The standard deviation of the Gaussian filter is 1.0. The mask of the median filter is composed of 7 × 7 pixels. The AWMF and the DM filter are implemented according to the algorithms described in [8, 9]. In our despeckling method, the standard deviation of the Gaussian kernel for computing local diffusion tensors is 1.4. For each test image, three passes of the despeckling pipeline are performed to reduce speckles. Six test images, including two ultrasound images and four grey-level images, are filtered in the experiments. The ultrasound images are despeckled by using these filters. The resulted images are visually compared. The four grey-level images are contaminated by multiplicative noises and filtered by using these filters. We use PSNR and SSIM values [15] to evaluate the filtered results so that objective comparisons can be made. This section also presents computational cost analysis for the proposed method. The analysis shows that the time complexity of our despeckle method is roughly linear with respect to the data size.

### 3.1. Despeckled Results of the Ultrasound Data

**Figure 5:**
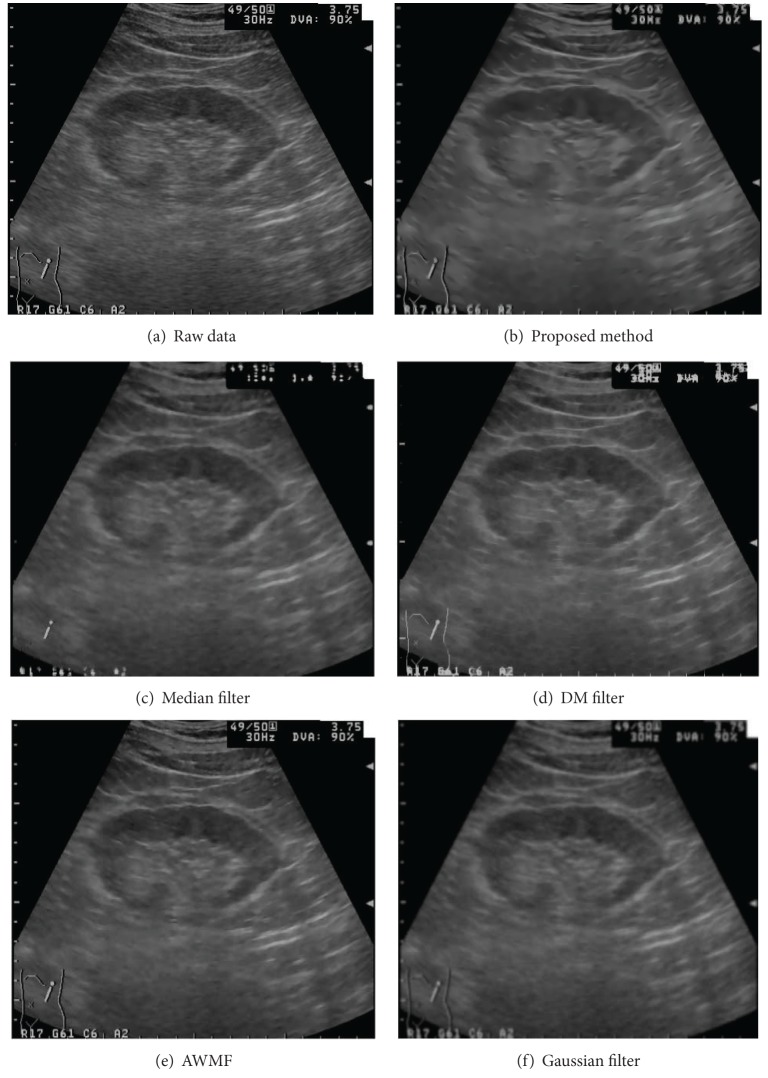
The despeckled results of the kidney data by using different filters.

**Figure 6:**
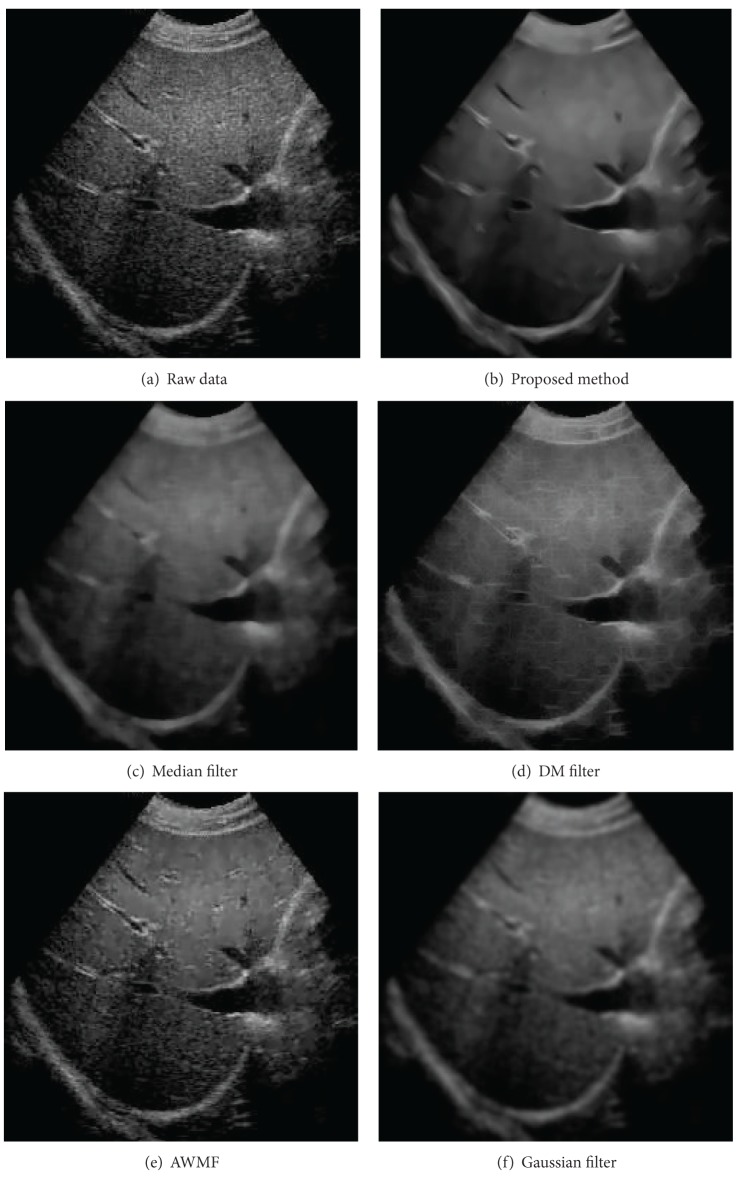
The despeckled results of the liver data by using different filters.

The ultrasound images are produced from ultrasound scanning of a kidney and a liver. The raw images and the despeckled results are shown in Figures 5 and 6. The raw kidney image is displayed in part (a) of Figure 5. The despeckled results of the 2nd pass of the proposed despeckle method are shown in part (b). The denoised results of the other filters are displayed in parts (c)–(f). The resultant images show that the Gaussian filter reduces the noise level but blurs edges. The median filter produces similar results, though it preserves more features. The DM filter sharpens edges. However, it generates honeycomb patterns in the resulted image. The AWMF method reduces less noise but preserves more edges. The proposed filtering method removes significant amount of speckles in homogeneous regions and enhances tissue boundaries. It produces the best results.

The raw liver image is shown in part (a) of Figure 6. The filtered results of the proposed filter and the other denoising methods are displayed in parts (b)–(f). Two passes of the proposed despeckling procedure are performed to reduce the speckles. The proposed filtering method produces the clearest results. It removes most speckles in smooth regions and enhances tissue boundaries. The median filter and the Gaussian filter suppress speckles, but they blur edges. The AWMF method reduces less noise, compared with the other filters. The DM filter produces artifact patterns while highlighting edges.

### 3.2. Multiplicative Noise Reduction

**Figure 7:**
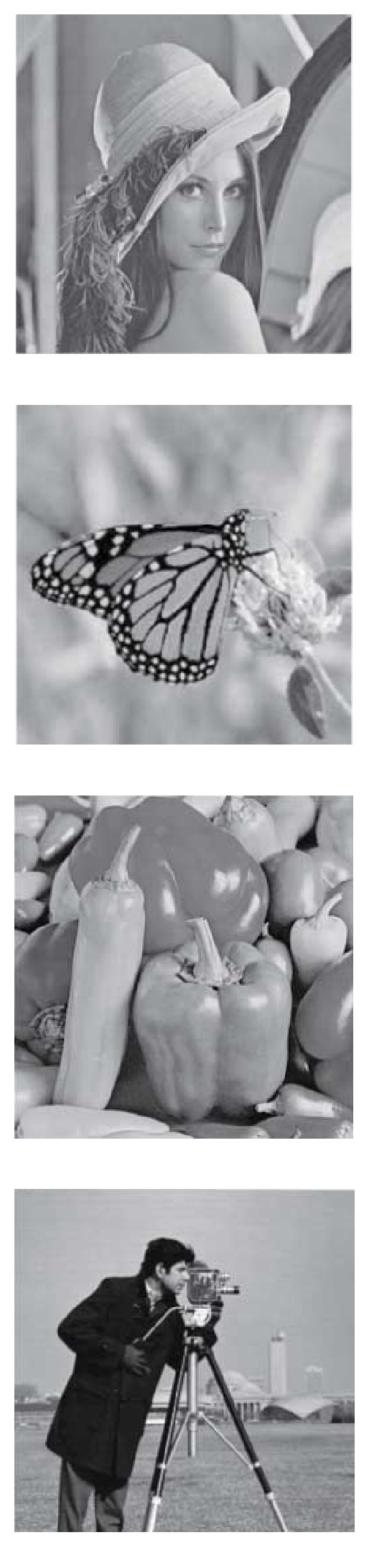
The grey-level images: Lena, butterfly, peppers, and cameraman.

In [1], speckles are modeled as multiplicative noise. In the second experiment, these despeckle methods are utilized to remove multiplicative noise hidden in four grey-level images. The grey-level images are shown in Figure 7. They are contaminated with Rayleigh noises with various standard deviations, *σ* = 0.1,0.2,0.3,0.4 and 0.5,

(8) $$\begin{array}{c} I^{*}\left(x,y\right)=I\left(x,y\right)*n\left(x,y\right), \end{array}$$

where *I*(*x*, *y*) represents the noise-free images and *n*(*x*, *y*) is the multiplicative Rayleigh noise. The noisy images are filtered by using these denoising methods. The PSNR and SSIM values of the noisy images and the filtered results are listed in Tables 2 and 3. The SSIM and the PSNR values of the noisy images are contained in the 2nd columns of these tables. Three passes of the proposed despeckle procedure are performed for each noisy image. The PSNR and SSIM values of the three filtering passes are listed in the 3rd columns. The two measurements of the other filters are contained in the 4th columns. Each row of these tables displays the PSNR and SSIM values for one noisy image with a fixed noise level.

#### 3.2.1. Comparisons Based on PSNR

The PSNR values are employed to measure the intensity differences of the noise-free images and the filtered results. Based on the data of Table 2, the proposed filtering method does increase the PSNR values for the test images. It produces the best PSNR values at the 1st filtering pass if the noise level is low (*σ* = 0.1). When *σ* ≥ 0.3, extra filtering passes improve the PSNR values. The Gaussian filter and the median filters improve the PSNR values in general. They increase the PSNR values for the first three test images with all noise levels. But, they decrease the PSNR value for the cameraman image with *σ* = 0.1. The AWMF method is very effective in removing noise for the images with the lowest noise level *σ* = 0.1. However, it produces the worst PSNR values when the noise level is higher. The DM filter is superior to the AWMF method but inferior to the other filters. Based on the results, we conclude that the proposed method produces the best PSNR values in most cases. The only exception is the cameraman image. It is inferior to the AWMF method if *σ* = 0.1. When *σ* = 0.2, the proposed method is lightly worse than the Gaussian filter.

#### 3.2.2. Comparisons Based on SSIM

The SSIM values listed in Table 3 are used to evaluate the visual perception quality of the filtered results. The SSIM values reveal that all the filters, except the AWMF method, significantly improve the perception quality for the contaminated images of all noise levels. For the least noisy images, *σ* = 0.1, the performance of the AWMF is competitive. However, it does not improve the SSIM values if the noise level is higher. The proposed filter method generates the best SSIM values at the 1st or the 2nd filtering pass when *σ* = 0.1. For the images with higher noise levels, the SSIM values are improved as extra filtering passes have been carried out. Compared with the other filters, the proposed method usually produces better SSIM values. The exceptions are the cameraman image and the peppers image with the noise level *σ* = 0.1. In these two cases, the AWMF and the Gaussian filter generate slightly better SSIM values.

#### 3.2.3. Comparisons Based on Appearances

**Figure 8:**
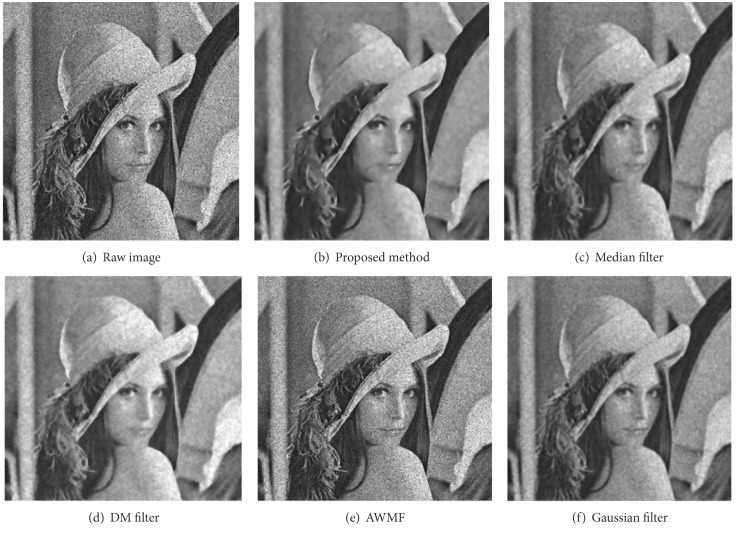
The filtered results of the Lena image.

**Figure 9:**
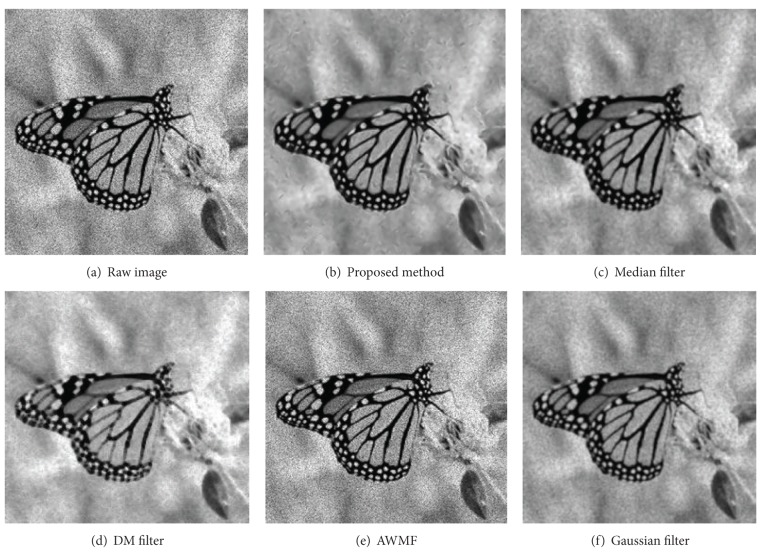
The filtered results of the butterfly image.

**Figure 10:**
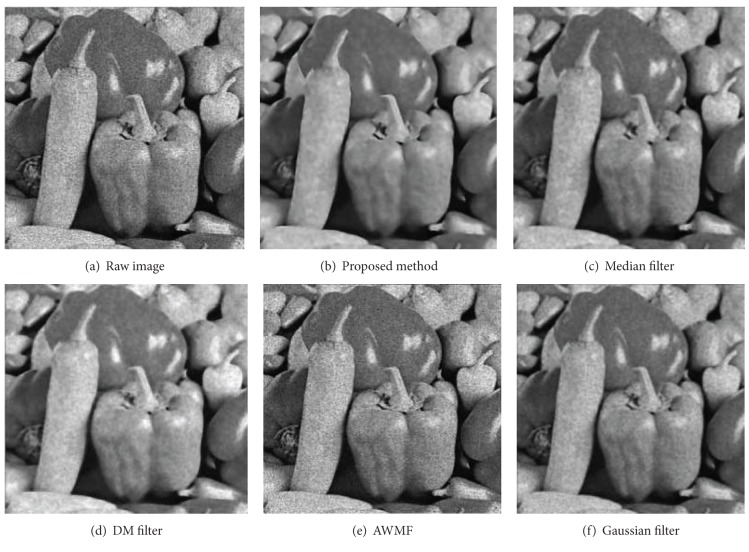
The filtered results of the peppers image.

**Figure 11:**
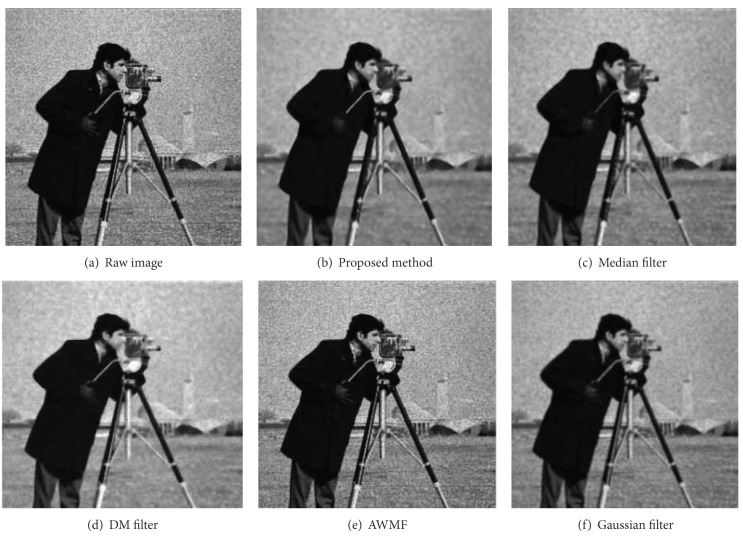
The filtered results of the cameraman image.

Figures 8, 9, 10, and 11 display partial filtered results of the second experiment. The noise level of the contaminated images is *σ* = 0.3. The noisy images are shown in parts (a) of these figures. The denoised results produced by the proposed method, the median filter, the DM filter, the AWMF method, and the Gaussian filter are contained in parts (b)–(f) of these figures. Two passes of the proposed filtering method are performed to produce the denoised images. According to the appearances of the resulted images, the AWMF produces the worst results for all the test images. In the AWMF implementation, we use the default constants listed in [9] to compute pixel weights for the median filter. It is obvious that these default constants are not feasible for filtering these test images. The DM filter preserves thin edges well, but it reduces less noise and adds artifact patterns to the resulted images (the cameraman and the butterfly images). The Gaussian filter suppresses noise and generates smooth results, but it blurs thin edges (the camera handle in the cameraman image) and region boundaries (the Lena image). The median filter is similar to the Gaussian filter. The proposed method preserves large-grain structures and reduces noise in smooth areas. It produces the cleanest results. Fine structures, which are brighter or darker than the surrounding areas, are enhanced (the butterfly wings in the butterfly image and the camera handle and tripod in the cameraman image). Fuzzy and thin structures may be blurred (the feather in the Lena image). Region boundaries are always well preserved and even enhanced (the peppers image and the butterfly image).

### 3.3. Computational Cost Analysis

The costs for performing one filtering pass of the proposed method are listed in Table 4. The titles and sizes (in pixels) of the images are shown in the 1st and 2nd columns. The costs for equalizing the raw images, computing Hessian matrices and eigensystems, classifying structure types, and filtering pixels are contained in the 3rd–6th columns. The costs are measured by seconds. The numbers contained in the parentheses are the percentages of these costs in relation to the total costs. The total costs are displayed in the last column. The embedded machine is a desktop computer equipped with a 2.93 GHz dual-core CPU and 3.2 Gbyte memory. The cost breakdown in the table indicates that computing Hessian matrices and eigensystems is the most expensive step. It may comprise more than 50% of the total cost. The filtering step spends about 30% of the total costs in average. The equalization process constitutes 20% of the total costs. The costs of the equalization process and the computation of Hessian matrices and eigensystems are linearly dependent on the image size. Since they make up about 70% of the total cost, the time complexity of the proposed method is roughly linear with respect to the input data size.

## 4. Conclusion

In this paper, we present a despeckle pipeline for ultrasound data. Our method explores local structure information and roughness by using the diffusion tensor of intensity. To improve the classification, a refinement strategy is developed to eliminate scattering spots and produce larger regions and longer boundaries. Then, based on the computed structure types, feasible filters are selected from a filter pool to suppress speckles and enhance features. Based on the statistical and visual results presented in Sections 3.1 and 3.2, the proposed method is capable of reducing speckles in homogeneous regions, preserving edges, and enhancing region boundaries in heterogeneous regions. It is superior to the Gaussian filter and the 3 median filters. We also conduct objective comparisons between these filters by using gray-level images. The experimental results reveal that the proposed filter is also very effective in removing multiplicative noises for grey-level images.

## Figures and Tables

**Table 1:** Preliminary type classification rules.

Types	||*λ*_1_||	||*λ*_2_||
Uniform	Small	Small
Tubular	Large	Small
Spot	Large	Large

**Table 2:** PSNR of the noisy images and the denoised images by using different filters.

Images	Noisy image	Proposed method			Other filters			
		Pass 1	Pass 2	Pass 3	Gauss	AWMF	DM	Median
Lena								
*σ* = 0.1	25.7	32.4	31.8	31.0	29.7	30.6	27.4	28.6
*σ* = 0.2	19.8	27.2	29.6	29.8	28.5	19.9	24.2	27.3
*σ* = 0.3	16.6	26.2	27.7	27.9	26.9	16.6	21.6	26.0
*σ* = 0.4	14.3	24.1	26.1	26.6	25.4	14.3	19.6	24.8
*σ* = 0.5	12.8	22.0	24.4	25.1	24.0	12.8	18.1	23.8
Butterfly								
*σ* = 0.1	24.5	32.5	31.9	30.0	30.9	30.8	24.9	28.3
*σ* = 0.2	18.7	26.1	28.1	28.0	28.0	18.7	22.5	26.9
*σ* = 0.3	15.4	25.2	26.6	26.9	26.9	15.4	20.2	25.3
*σ* = 0.4	13.3	22.4	24.8	25.0	24.9	13.2	18.3	23.9
*σ* = 0.5	11.8	20.4	22.9	23.8	23.3	11.8	16.8	22.7
Peppers								
*σ* = 0.1	25.9	29.5	29.3	28.9	27.7	28.8	26.5	28.1
*σ* = 0.2	20.0	26.5	28.1	28.2	26.9	20.1	23.9	27.0
*σ* = 0.3	16.8	26.0	27.0	27.1	26.0	16.8	21.6	25.9
*σ* = 0.4	14.6	24.2	25.7	26.0	24.8	14.6	19.6	24.7
*σ* = 0.5	13.0	22.8	24.6	25.1	23.7	13.0	18.1	23.8
Cameraman								
*σ* = 0.1	25.6	26.7	26.3	25.8	24.8	28.7	24.0	24.0
*σ* = 0.2	19.7	24.3	24.3	24.1	24.4	19.7	22.3	23.5
*σ* = 0.3	16.4	23.4	23.7	23.7	23.5	16.4	20.4	22.9
*σ* = 0.4	14.3	22.5	23.1	23.1	23.0	14.3	18.7	22.1
*σ* = 0.5	12.8	21.6	22.3	22.4	22.2	12.8	17.4	21.4

**Table 3:** SSIM of the noisy images and the denoised images by using different filters.

Images	Noisy image	Proposed method			Other filters			
		Pass 1	Pass 2	Pass 3	Gauss	AWMF	DM	Median
Lena								
*σ* = 0.1	0.8111	0.9238	0.9255	0.9128	0.9184	0.9088	0.8976	0.8798
*σ* = 0.2	0.6158	0.7498	0.8309	0.8631	0.8243	0.6221	0.7970	0.8189
*σ* = 0.3	0.4900	0.5964	0.8006	0.8194	0.7294	0.4902	0.7019	0.7496
*σ* = 0.4	0.4033	0.4868	0.7309	0.7730	0.6508	0.4033	0.6271	0.6819
*σ* = 0.5	0.3538	0.4166	0.6543	0.7071	0.5899	0.3458	0.5678	0.6237
Butterfly								
*σ* = 0.1	0.7312	0.9425	0.9511	0.9448	0.9289	0.8858	0.8888	0.9214
*σ* = 0.2	0.5041	0.8502	0.8913	0.8975	0.8197	0.5047	0.7749	0.8376
*σ* = 0.3	0.3909	0.7214	0.7931	0.8194	0.7112	0.3909	0.6633	0.7389
*σ* = 0.4	0.3270	0.5988	0.6751	0.7060	0.6220	0.3270	0.5735	0.6491
*σ* = 0.5	0.2877	0.5065	0.5780	0.6724	0.5573	0.2877	0.5116	0.5735
Peppers								
*σ* = 0.1	0.8353	0.9103	0.9091	0.9012	0.9128	0.8930	0.8842	0.8895
*σ* = 0.2	0.6369	0.8074	0.8676	0.8802	0.8467	0.6397	0.8150	0.8404
*σ* = 0.3	0.5044	0.7181	0.7822	0.8034	0.7734	0.5045	0.7383	0.7804
*σ* = 0.4	0.4141	0.6505	0.7317	0.7800	0.7208	0.4142	0.6671	0.7166
*σ* = 0.5	0.3556	0.6479	0.7237	0.7522	0.6463	0.3556	0.6111	0.6646
Cameraman								
*σ* = 0.1	0.7891	0.8494	0.8342	0.8187	0.8567	0.8575	0.8142	0.7885
*σ* = 0.2	0.6117	0.7006	0.7442	0.7619	0.7525	0.6102	0.7096	0.7236
*σ* = 0.3	0.5162	0.6741	0.7166	0.7301	0.6965	0.5160	0.6348	0.6536
*σ* = 0.4	0.4555	0.6135	0.6594	0.6784	0.6412	0.4556	0.5724	0.5933
*σ* = 0.5	0.4084	0.5568	0.6020	0.6212	0.6212	0.4084	0.5246	0.5370

**Table 4:** Cost breakdown of the proposed method, measured by seconds. The numbers in parentheses are percentages.

Images	Size	Equal. (%)	*λ*_1_, *λ*_2_ (%)	Class. (%)	Filter. (%)	Total
Lena	512 × 512	0.60 (20.6)	1.41 (48.3)	0.01 (0.3)	0.90 (30.8)	2.92
Butterfly	512 × 512	0.60 (21.1)	1.39 (48.9)	0.03 (1.1)	0.82 (28.9)	2.84
Peppers	512 × 512	0.61 (20.3)	1.40 (46.7)	0.02 (0.7)	0.97 (32.3)	3.00
Cameraman	512 × 512	0.64 (20.9)	1.40 (45.8)	0.01 (0.3)	1.01 (33.0)	3.06
Kidney	469 × 379	0.39 (20.7)	0.95 (50.5)	0.01 (0.5)	0.53 (28.2)	1.88
Liver	246 × 235	0.11 (20.4)	0.31 (57.4)	0.00 (0.0)	0.12 (22.2)	0.54
